# YOUNG ADULTS STEREOTYPE OLDER SPEAKERS WHO ADOPTED A POWER POSE AS LESS COMPETENT COMPARED TO SUBMISSIVE OR CONTROL

**DOI:** 10.1093/geroni/igz038.310

**Published:** 2019-11-08

**Authors:** Jennifer R Turner, Jennifer T Stanley

**Affiliations:** University of Akron, Akron, Ohio, United States

## Abstract

Young adults (YA) frequently endorse age stereotypes (Levy, 2009). We examined whether older adult (OA) speakers influenced by embodied-cognition (“power posing”; Cuddy et al., 2015) would reduce YAs’ stereotype-related judgments. Following the Stereotype Content Model (SCM; Fiske et al., 2002), we hypothesized that OA who held a power pose prior to giving their speech would be rated as higher in Competency, Performance, and Electability, but not Warmth. Sixty-three YA viewed and rated 9 videos of OA performing speeches after modeling a pose (power, submissive, control). Within-subjects ANOVAs revealed embodiment condition differences for Performance (F2,124 = 207.76, ηp2 = .77). For ratings of Performance, speakers in the power condition were judged worse than either submissive or control (ps < .001). For Warmth ratings, power (M = 4.81, SD = .62) was worse than control (M = 5.07, SD = .89, p = .003, d = .34), but submissive (M = 4.97, SD = .87) was not significantly different from either group. These results suggest that YA may judge the Performance and Warmth of OA who adopted a power pose harsher because OA are not supposed to be powerful or adopt expansive postures (consistent with the SCM). In comparison, YA may be drawing upon the Representativeness Heuristic of OA in positions of power (e.g., Senators) when rating Electability and Competence.

